# Nano Zinc Oxide Improves Performance, IGF-I mRNA Expression, Meat Quality, and Humeral Immune Response and Alleviates Oxidative Stress and NF-κB Immunohistochemistry of Broiler Chickens

**DOI:** 10.1007/s12011-022-03494-y

**Published:** 2022-11-26

**Authors:** Heba A. Alian, Hayam M. Samy, Mohammed T. Ibrahim, Mohamed S. Yusuf, Manal M. A. Mahmoud

**Affiliations:** Faculty of Veterinary Medicine, Department of Nutrition and Clinical Nutrition, Suez Canal University, Ismailia, 41522 Egypt

**Keywords:** Antibody titer, Antioxidant, Broilers, Insulin-like growth factor I, Meat quality, Nuclear factor kappa B

## Abstract

A 35-day trial was set to explore the effects of different dietary zinc sources on growth, insulin-like growth factor I (IGF-I) mRNA expression, meat quality, immune response, antioxidant activity, and immunohistochemistry of nuclear factor kappa B (NF-κ7B) of broiler chickens. Ross 308 broiler chicks (*n* = 156) were randomly assigned into four experimental groups. The G1 received the basal control diet without zinc supplementation; the G2, G3, and G4 were supplemented with zinc oxide, zinc lysine, and nano zinc oxide, respectively, at a level of 40 mg Zn/kg diet. The data revealed that nano zinc oxide linearly improved the overall growth performance parameters. Nano zinc oxide linearly elevated (*P* < 0.001) mRNA expression of IGF-I followed by G3. The pH value of breast muscle in G4 shows a linearly decreasing value (*P* < 0.001). Also, the linearly highest expressible release volume percentage and lightness (L*) value with the lowest redness (a*) value (*P* < 0.05) were recorded in G4 and G3. A numerical increase in the total antibody titer was recorded on the 35^th^ day in the G3 and G4. A numerical elevation in the superoxide dismutase (SOD) and a numerical reduction in the serum malondialdehyde (MDA) were recorded in the G4. The section of the liver from G4 revealed significantly very low expression of NF-κB staining. It is concluded that nano zinc oxide is considered the more trending zinc source. It had no negative effects on the health status and can be used in broiler diet premix.

## Introduction

Poultry meat production and consumption have increased rapidly worldwide and are predicted to continue to rise [1], because of its low cost compared to other meats, lack of cultural or religious barriers, and nutritional value [2]. Therefore, improving poultry production is one of the main goals of both the private and the public sectors. There is a growing concern about how to improve the effectiveness of trace element supplementation in broiler diets on growth, productivity, and reproductive performance. Recently, zinc (Zn) nutrition has become a monumental task at the forefront of many researchers’ minds. By way of example, zinc is a crucial element that acts as a cofactor in various metabolic processes essential for hormone secretion, i.e., insulin and growth hormones [3], DNA synthesis, gene expression, and cellular division [4]. The zinc level in the raw materials of the diet is insufficient to meet the needs of animals. Moreover, Zn cannot be stored in an animal’s body; hence, there is a need for daily supplementation of Zn through the diet [5].

Many researchers have added Zn to broiler diets in inorganic [6, 7] or organic [8, 9] form and observed an improvement in growth performance. However, in monogastric animals, the bioavailability of inorganic zinc is much lower [10]. Thence, organic sources may be used to replace inorganic sources of trace minerals to reduce over-supplementation and their excretion [11]. Organic sources of zinc, i.e., Zn lysine and Zn methionine, have been found to substitute inorganic sources due to their higher bioavailability without having a harmful effect on poultry production and the environment [12]. Despite this, organic Zn supplementation in broiler diets has been limited due to its relatively high cost. Thus, to get better Zn bioavailability, other approaches should be considered. However, as broiler strains continue to be genetically modified, the zinc requirement of NRC [13], 40 mg/kg of diet is not adequate to support the growth rate, health status, and reproduction of modern broiler strains [14]. Hence, zinc supplementation in the diet is at levels that are two to 10 times higher than those recommended by the NRC [15]. Recently, nanotechnology has been extensively applied in animal nutrition to enhance the utilization of trace elements. Nano zinc oxide (NZnO) is considered one such nanotechnological trail that is characterized by strong catalytic efficacy and high adsorbing capability [16]. In addition to being highly bio-available, studies have already pointed out the growth enhancement, antibacterial activity, and modulation of the immunity and reproduction of animals, among many more effects of nano zinc [17]. Studies comparing inorganic zinc, organic zinc, and nano zinc oxide supplementation as feed additives in broiler chicken diets are limited. Accordingly, this experiment aimed to evaluate the effects of different dietary Zn sources on growth, mRNA gene expression of IGF-I, meat quality, immune response, antioxidant status, and immunohistochemistry of nuclear factor kappa B of broiler chickens.

## Materials and Methods

The procedures of this work were performed and authorized by the strategies of the animal care and ethics committee of the Faculty of Veterinary Medicine, Suez Canal University, Egypt (approval no. 2019040).

### Experimental Birds and Management

One-day-old unsexed Ross 308 broiler chicks (*n* = 156) were purchased from a private company in Ismailia Province, Egypt. The chicks were reared in an environmentally controlled place. During the first week of life, the temperature was at 32–34 °C; after that, it dropped by 2 °C/week until the third week. The lighting program in the first week was 23 h of light and one hour of darkness, followed by two hours of darkness until 35 days of age. The chicks were allowed to have free access to water and feed. The broiler chicks were housed in a designed battery system with semi-automated feeders and drinkers; each replicate cage had a one-meter square area (50-cm long × 200-cm wide × 50-cm height). The birds were followed at 7 am, 3 pm, and 11 pm for water, feed, and mortality. On days 9 and 24, all the chicks received vaccinations for Newcastle disease; on day 14, they were vaccinated for the infectious bursal disease. All management procedures were carried out based on the Ross 308 Broilers Management Guide [18].

### Experimental Design

The birds were randomly distributed into four experimental groups. These groups were subdivided into three replicates (each of 13 birds). The experimental groups were as follows: the G1 as a control group received the basal diet without zinc supplementation. The G2 was supplied with zinc oxide (LOBA Chemie Co. product analysis Art.06550, ZnO) as an inorganic Zn source. The G3 was supplied with zinc–lysine (Availa zinc 170®, batch number: CPD 21,050 produced by Multi Vita Co. for animal nutrition, October City, Egypt), as a Zn organic source. The G4 was supplied with a prepared nano zinc oxide (NZnO). Following a zinc analysis of the basal diet (Table 1), the required amount of zinc oxide was added to reach the bird’s requirement (40 mg Zn/kg diet), followed by the zinc supplementary level in G2, G3, and G4 by 40 mg Zn/kg diet.

**Table 1:** The basal diet composition with calculated chemical analysis (1–5 weeks)^a^

Ingredients (%)	Starter (0–10 d)	Grower (11–24 d)	Finisher (25–35 d)
G. yellow corn (8.7% CP)^b^	55.592	58.900	64.212
Soya bean meal (45.3% CP)^b^	32.360	28.200	22.292
Corn gluten (63.7% CP)^b^	5.500	5.708	5.990
Soybean oil	2.630	3.560	4.100
Dical. phosphate (22% Ca &19% P)	1.494	1.300	1.150
Limestone (38% Ca)	1.363	1.270	1.200
L–lysine (purity 99%)	0.300	0.318	0.330
DL–methionine (purity 99%)	0.161	0.144	0.126
Mineral & vitamins premix ^c^	0.300	0.300	0.300
Iodized sodium chloride	0.300	0.300	0.300
Total	100.000	100.000	100.000
Calculated composition			
Crude protein (%)	23.000	21.500	19.500
ME (Kcal per kg)	3000.000	3100.000	3200.000
Calorie/protein ratio (C/P)	130.430	144.180	164.100
Calcium (%)	0.960	0.870	0.790
Phosphorus (%)^d^	0.300	0.435	0.395
Zn (mg/kg)^e^	18.030	10.650	12.810
Still need Zn (mg/kg)^f^	21.970	29.350	27.190
Added amount of ZnO (mg/kg)^g^	27.350	36.530	33.840

^a^Prepared according to nutrient requirement of Ross 308 (Aviagen, 2019)

^b^Chemical analysis according to (AOAC, 2002)

^c^The following nutrients are included in each 3 kg: vit. A 12 mIU, vit. E 10,000 mg, vit. D3 2 mIU, vit. k3 2,000 mg, vit. B1 1,000 mg, vit. B2 5,000 mg, vit. B6 1,500 mg, vit. B12 10 mg, pantothenic acid 10,000 mg, biotin 50 mg, folic acid 1,000 mg, nicotinic acid 30,000 mg, manganese oxide 60,000 mg, zinc oxide 50,000 mg, copper sulfate 10,000 mg, iron sulfate 30,000 mg, potassium iodide 1,000 mg, cobalt sulfate100 mg, sodium selenate 100 mg; CaCO_3_ was a carrier up to 3 kg (Avimix for broilers premix- AGRIVET Co, Elasher, Egypt. patch No. 2321, production 25–4-2021)

^d^Available phosphorus

^e^Analysis was conducted at Central lab, Faculty of Veterinary Medicine, Zagazig University, Egypt

^f^The amount of zinc required to meet broiler zinc requirements (40 mg Zn/kg diet)

^g^The calculated amount is based on each 100 mg of zinc oxide containing 80.34 mg of zinc

### Nano Zinc Oxide Preparation and Characterization

**Fig. 1:**
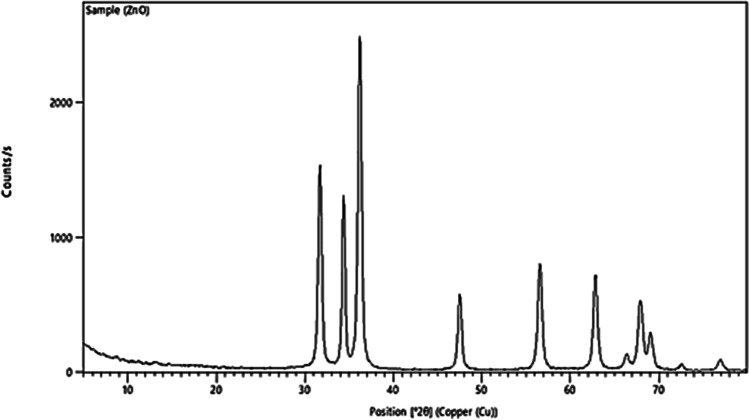
Nano zinc oxide characterization by XRD techniques

**Fig. 2:**
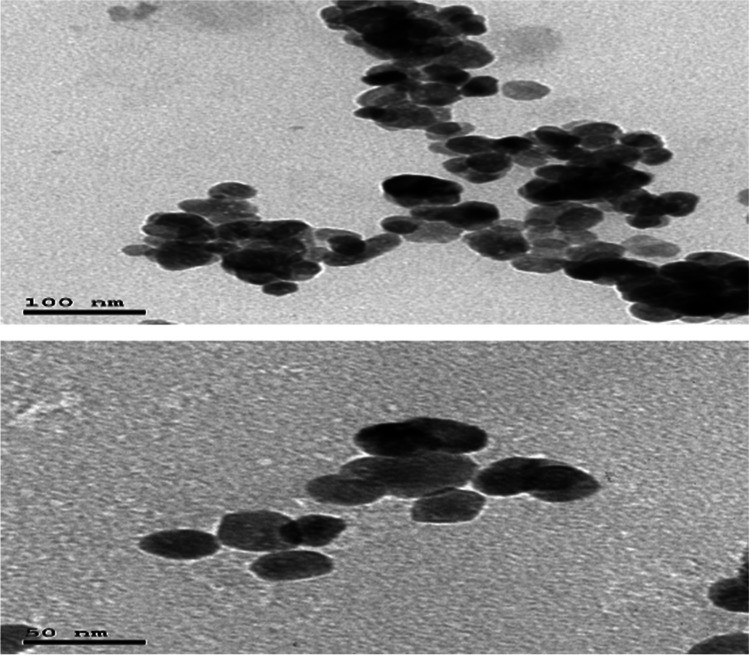
Nano zinc oxide characterization by HRTEM techniques. HRTEM image of nano ZnO at different scales (100 nm and 50 nm)

Nano zinc oxide has been synthesized as stated by Beek et al. [19] and Pacholski et al. [20], via potassium hydroxide hydrolyzing and condensing zinc acetate dihydrate in an alcoholic medium at low temperatures. After removing the surplus mother liquor and washing the precipitate with methanol, the NZnO was collected at the bottom. The precipitate was then mixed with methanol and chloroform to dissolve it (preparation was done at Nano Gate Co. for Synthesizing of Nanomaterials, Nasr City, Egypt). The nanoparticles were evaluated by X-ray diffraction (XRD). Figure 1 displays the XRD diagram of the NZnO, revealing that the prepared sample is in nano size and well-coordinated with the standard data from the JCPDS card (Joint Committee on Powder Diffraction Standards). Also, the sample was characterized by a high-resolution transmissible electron microscope (HRTEM). Figure 2 showed that the prepared nanoparticles are uniform in shape and size with no aggregation, representing homogeneity. The HRTEM image displayed the size of NZnO was about 30 ± 5 nm.

### Experimental Diets

Three experimental corn–soybean-based diets were formulated (Table 1) to supply the nutrient requirements of Ross 308 broiler chicks according to Aviagen [21]. The experimental diets were made in pellet form with different sizes according to the bird's age by a feed pelleting machine. Proximate chemical analysis of the experimental diets and feed ingredients and the zinc analysis of basal diets using a flame atomic absorption spectrophotometer were done according to the standard methods described in AOAC [22].

### Growth Performance Parameters

Body weight (BW) and chicks’ weight gain (WG), feed intake (g), feed conversion ratio (FCR), and feed efficiency (FE) for each replicate were measured weekly according to Eskandani et al. [23]. According to El-Katcha et al. [24], the performance index (PI) was determined as live BW (kg)/FCR × 100, the European efficiency index (EEI) was calculated according to Eskandani et al. [23] as 100 × ( BW (kg) × livability (%)/(age (days) × FCR)), and the protein efficiency ratio (PER) was determined as WG (g)/protein intake (g).

### Sampling

On the 35^th^ day of the experiment, two birds per replica were randomly chosen, and only water was allowed overnight. Each bird was weighed and then slaughtered by manual slaughtering technique and subjected to a meat quality assessment. Blood samples were collected with anticoagulants and then centrifuged for 15 min at 3000 rpm, and serum was collected and kept at -20 °C until further analysis. Tissue samples of the liver of the same slaughtered birds were gathered and saved for histopathological and immunohistochemical examination. For mRNA gene expression testing, a portion of the liver tissue was removed and stored at − 80 °C.

### mRNA Gene Expression of IGF-I in Liver Tissues

The QIAamp RNeasy Mini kit (Qiagen, Germany, GmbH) was used to extract RNA from liver tissue samples, with 30 mg of liver sample added to 600 µl of RLT buffer containing 10 µl of mercaptoethanol per 1 ml. Tubes were inserted into adapter sets, which are fitted into the clamps of the Qiagen tissue Lyser, to homogenize the samples. Disruption was completed in a 2-min high-speed (30 Hz) shake step. One volume of 70% ethanol was mixed with the cleared lysate, and the steps were performed based on the Purification of Total RNA from Animal Tissues protocol of the QIAamp RNeasy Mini kit (Qiagen, Germany, GmbH). The primers were obtained by Metabion (Germany) and are listed in Table 2. The reaction was done on a Stratagene MX3005P real-time PCR machine. The Stratagene MX3005P software calculated the Ct values and amplification curves. To test the difference in RNA gene expression between the samples, the CT of each sample was compared with the positive control group according to the “ΔΔCt” method described by Yuan et al. [25] using the 2^−ΔΔct^ ratio.

**Table 2:** Primer sequences of target genes

Target gene	Primer sequences (5′-3′)
*ß-actin*	CCACCGCAAATGCTTCTAAAC
	AAGACTGCTGCTGACACCTTC
*IGF1*	CAACGAGCGGTTCAGGTGT
	TGGAGTTGAAGGTGGTCTCG

### Meat Quality Parameters

Breast fillets of birds (two from each replicate) were evaluated for the meat quality as follows: the pH was measured by homogenizing 10 g of breast muscle with 90 ml of distilled water for 30 s, and then, the homogenates were obtained. The pH of each sample was measured with a pH meter (ADWA, AD 11) at 25 °C [26]. After every five measurements, the pH meter was calibrated using standard pH solutions (pH = 4 and 7). Color measurements were taken using a chromameter (Minolta Camera Co. Ltd., Osaka, Japan) that was previously standardized. Color for each sample was stated in terms of CIE (The International Commission on Illumination) values for lightness (L*), redness (a*), and yellowness (b*) as mentioned by Shim et al. [27]. Expressible release volume (ERV) and water-holding capacity (WHC) % were determined according to the technique stated by Wilhelm et al. [28], it was done by cutting 5 g samples (initial weight) into smaller pieces and then pressing them with the 2500 g weight for 5 min within two filter papers (15.0 cm circles, fine crystalline retention). The samples were removed from the filter paper, and their weight was measured (final weight). ERV was defined as water loss and determined as follows: ((initial weight − final weight)/ initial weight) × 100. Therefore, a lower percentage of ERV indicated a greater WHC [29]. The hardness (kg/cm^2^) of *breast* fillets was measured by a firmness meter tester after 24 h. according to the method used by Volovinskaia and Kelman [30]. For drip loss percentage determination, the breast muscles were weighed and then put in Ziploc bags and kept at 4 °C for 24 h. Then, it was measured as the percentage of the difference between the weights before and after chilling for 24 h and divided by the first weight as stated by Saenmahayak et al. [31].

### Humeral Immune Response, Hemagglutination Inhibition (HI) Test for Determination of Antibody Titer Against Live Newcastle Disease (ND) Vaccination

Blood samples were collected at zero, 23^rd^, and 35^th^ days of age from three birds in each group. Thereafter, an identical amount of 4 HA units of the ND virus was produced and added to each serial two-fold serum dilution of the collected sera (50 µl). Then, 50 µl of 0.5% washed chicken red blood cells (RBCs) was added to each well after 30 min of incubation. The blend was incubated for 30 min at room temperature. HI endpoints was the highest dilution of serum that makes complete inhibition of viral hemagglutination. HI endpoints were recorded according to the OIE [32] technique.

### Oxidative Stress Biomarkers

Superoxide dismutase (SOD) activity was measured using a quantitative colorimetric method with a ready-made kit, as described by Ōyanagui [33]. The malondialdehyde (MDA) was determined by the quantitative colorimetric method using a ready-made kit according to the method stated by Ohkawa et al. [34]. SOD and MDA content were measured using Bio-Diagnostic Company, Cairo, Egypt reagent kits as per manufacture procedures.

### Immunohistochemistry (IHC) of Nuclear Factor Kappa B in Liver Tissues

Liver tissue samples (5 μm) were put in 4% formaldehyde and then processed on positively charged slides for NF- κ B immunohistochemical detection. Samples were embedded in paraffin, then deparaffinized in xylene (twice), and immersed in a series of descending concentrations of absolute, 95%, 70%, and 30% ethanol, followed by water. After blocking endogenous peroxidase activity with 1% H_2_O_2_, the antigenicity was unmasked by 3 min of microwave heating in a 10-mM sodium citrate solution. A blocking solution of 2% dry milk in PBS (containing 0.02 percent sodium azide) was also used to reduce non-specific staining.

In a humidified chamber, sections were incubated for 2 h at 25 °C with a dilution of 1: 300 of a primary rabbit polyclonal anti-NF-kB/p65 antibody (Cat. No. GTX102090, GeneTex, Inc., North America). Following incubation, sections were washed three times with PBS for three minutes each, and liver sections were co-incubated for 30 min with a biotinylated polyvalent secondary antibody (Thermo Scientific Co., UK). After incubation, slides were washed three times with wash buffer for three minutes before being counterstained with enough hematoxylin stain to completely cover the tissue surface.

The intensity of the immuno-stained area (ISA) was quantitatively analyzed using an image analyzer (ImageJ program, Japan) [35].

### Statistical Analysis

A statistical software program (IBM SPSS statistics 21 for Windows, USA) was used to statistically analyze the obtained findings from experimental groups in comparison to the control group for the mean and standard error. Variances among means of experimental groups were carried out using ANOVA (one-way analysis of variance) with Duncan multiple comparison tests [36]. The significance between all groups was displayed using a *P* value < 0.05.

## Results

### Growth Performance Parameters

Nano zinc oxide (G4), 40 mg Zn/kg diet, linearly improved (*P* < 0.001) final body weight and cumulative body weight gain compared to G1, G2, and G3 (Table 3). The zinc lysine group, 40 mg Zn/kg diet, achieved an improvement in final body weight and cumulative weight gain linearly (*P* < 0.001) compared to chicks in G1. On the other hand, no statistical (*P* > 0.05) differences in the final body weights and the cumulative weight gains between experimental groups G1 and G2. Nano zinc oxide (G4) linearly achieved the best results in overall FCR, and FE improvement (*P* < 0.001) compared to chicks in G1, G2, and G3. Furthermore, the cumulative feed intake of G4 was linearly decreased (*P* = 0.003) compared to chicks in G1. Our results also revealed that zinc lysine (G3) and zinc oxide (G2) displayed linearly (*P* < 0.001) the best results in the FCR and FE with linearly lower feed intake (*P* = 0.003) compared to chicks in G1. Besides, nano zinc oxide at a level of 40 mg Zn/kg linearly gave (*P* < 0.001) the best result in the performance index (PI) compared to chicks in G1, G2, and G3. Nano zinc oxide linearly increased (*P* = 0.003) the European Efficiency Index (EEI) compared to chicks in G1 and G2 and numerically compared to G3. Nano zinc oxide linearly (*P* = 0.002) gave the best results in PER compared to chicks in G1, G2, and G3 during finisher phase.

**Table 3:** Impact of different zinc sources on growth performance parameters of broiler chicks at the end of the trail period (35 d)

	Group					Contrast *P* value	
Parameter	G1	G2	G3	G4	SEM*	Linear	Quadratic
Initial body weight (g)	46.21	45.19	46.22	45.93	0.18	0.913	0.267
Final body weight (g)	2263.66^c^	2280.93^bc^	2333.46^b^	2406.00^a^	18.31	< 0.001	0.155
Cumulative weight gain (g)	2217.45^c^	2235.73^bc^	2287.24^b^	2360.07^a^	18.31	< 0.001	0.163
Cumulative feed intake (g)	3240.97^a^	3118.13^b^	3088.01^b^	3033.16^b^	27.29	0.003	0.360
Feed conversion ratio	1.46^a^	1.39^b^	1.35^b^	1.28^c^	0.02	< 0.001	0.952
Feed efficiency	0.68 ^c^	0.71^b^	0.74^b^	0.77^a^	0.01	< 0.001	0.780
Performance index	154.87^d^	163.61^c^	172.80^b^	187.29^a^	3.78	< 0.001	0.299
European efficiency index	431.45^b^	467.47^b^	480.96^ab^	521.71^a^	11.68	0.003	0.880
Protein efficiency ratio (starter)	4.00	4.08	4.16	4.15	0.03	0.133	0.540
Protein efficiency ratio (grower)	3.35^c^	3.52^b c^	3.69^ab^	3.85^a^	0.06	0.002	0.944
Protein efficiency ratio (finisher)	2.96^b^	3.10^b^	3.14^b^	3.40^a^	0.05	0.002	0.351

^*^SEM, standard error of the mean; *G1*, basal diet only; *G2*, basal diet + zinc Oxide (40 mg Zn/kg diet); *G3*, basal diet + zinc lysine (40 mg Zn/kg diet); *G4*, basal diet + nano zinc oxide (40 mg Zn/kg diet)

Means in the same row with distinct superscripts are significantly different at *P* < 0.05

### mRNA Gene Expression of Insulin-Like Growth Factor I (IGF-I)

**Fig. 3:**
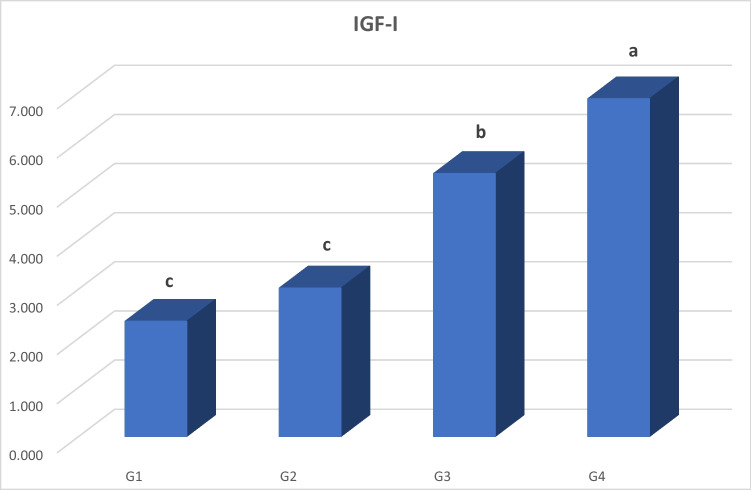
Gene expression of insulin-like growth factor I (IGF-I) in the liver of broiler chicks. Means having the separate letters are significantly dissimilar from each other, *P* < 0.05. G1: basal diet only. G2: basal diet + zinc oxide (40 mg Zn/kg diet). G3: basal diet + zinc lysine (40 mg Zn/kg diet). G4: basal diet + nano zinc oxide (40 mg Zn/kg diet)

Our findings showed that nano zinc oxide, 40 mg Zn/kg diet, linearly elevated (*P* < 0.001) mRNA expression of IGF-I in the liver tissues of broilers compared to chicks in G3, G2, and G1. Compared to the control, zinc lysine (G3) at a level of 40 mg Zn/kg diet linearly increased (*P* < 0.001) mRNA expression of IGF-I in the liver tissues of broilers, followed by the G2 that supplemented with zinc oxide at a level of 40 mg Zn/kg diet. Besides, the mRNA expression of the IGF-I gene tended to be linearly increased (*P* < 0.001) in G3 than in G2 (Fig. 3).

### Meat Quality Parameters

The impact of supplemental Zn on the meat quality traits of broilers is given in Table 4. There were linear and quadratic effects in the pH value of breast muscle in the nano zinc oxide-supplemented group, showing a linearly reducing value (*P* < 0.001) compared to G1, G2. Also, the linearly highest ERV% in breast muscle was recorded in birds receiving diets supplemented with nano zinc oxide and zinc lysine at 40 mg Zn/kg compared to G1 (*P* = 0.005). Furthermore, chickens in G4 and G3 had the highest linearly (*P* < 0.01) L* value in breast muscle. G4 and G3 had the lowest a* values (*P* < 0.05) when compared to G1 and numerically with G2. However, the b* value was not significantly affected (*P* = 0.259) by adding zinc sources in any group. No significant differences (*P* > 0.05) were noticed in hardness and drip loss percentages among experimental groups.

**Table 4:** Impact of different zinc sources on meat quality traits of broiler chicks

	Group					Contrast *P* value	
Parameter	G1	G2	G3	G4	SEM*	Linear	Quadratic
pH	6.83^a^	6.03^b^	5.80^bc^	5.56^c^	0.15	< 0.001	0.026
Expressible release volume (ERV)%	24.04^b^	28.45^ab^	33.30^a^	35.02^a^	1.60	0.005	0.559
Lightness (L*)	51.83^b^	51.74^b^	54.08^a^	54.13^a^	0.40	0.009	0.929
Redness (a*)	2.43^a^	1.53^ab^	1.23^b^	1.15 ^b^	0.19	0.016	0.267
Yellowness (b*)	8.84	9.32	8.60	8.31	0.22	0.259	0.403
Hardness (kg/cm^2^)	2.92	3.06	2.85	2.65	0.08	0.179	0.319
Drip loss (%)	1.60	2.20	1.62	1.84	0.16	0.918	0.583

^*^SEM, standard error of the mean; *G1*, basal diet only; *G2*, basal diet + zinc oxide (40 mg Zn/kg diet); *G3*, basal diet + zinc lysine (40 mg Zn/kg diet); *G4*, basal diet + nano zinc oxide (40 mg Zn/kg diet)

Means in the same row with distinct superscripts are significantly different at *P* < 0.05

### Antibody Titer Against Live ND Vaccination

Our data clarified that maternal antibodies were high in all groups. The dietary concentration of different zinc sources (G2, G3, and G4) at a level of 40 mg Zn/kg diet did not influence (*P* = 1.00) the antibody titer against ND vaccine on the 23^rd^ day in broiler chicks compared to the control group. Though, a numerical increase in the total antibody titer was recorded on the 35^th^ day in the zinc lysine (G3) and nano zinc oxide (G4) groups compared to G1 and G2 (Table 5).

**Table 5:** Impact of different zinc sources on antibody titers against live Newcastle disease virus in broiler chicks

	Group					Contrast *P* value	
Day	G1	G2	G3	G4	SEM*	Linear	Quadratic
Zero day (maternal immunity)	6.2	6.2	6.2	6.2	0.21	1.00	1.00
23^rd^ day (first immune response)	1.33	1.33	1.66	1.33	0.25	0.904	0.789
35^th^ day (second immune response)	4.75	4.50	5.16	4.83	0.17	0.581	0.910

^*^SEM, standard error of the mean; G1: basal diet only; *G2*, basal diet + zinc oxide (40 mg Zn/kg diet); *G3*, basal diet + zinc lysine (40 mg Zn/kg diet); *G4*, basal diet + nano zinc oxide (40 mg Zn/kg diet)

Means in the same row with distinct superscripts are significantly different at *P* < 0.05

### Oxidative Stress Biomarkers

The highest numerical, non-significant, improvement in superoxide dismutase (SOD) activity was observed in G4, G3, and G2 compared to the control. Also, the highest numerical, non-significant, reduction in serum MDA was detected in the nano zinc oxide supplemented group compared with other groups (Table 6).

**Table 6:** Oxidative stress biomarkers of broiler chicks

	Group					Contrast *P* value	
Parameter	G1	G2	G3	G4	SEM ^*^	Linear	Quadratic
Superoxide dismutase (SOD) (U/ml)	522.66	547.00	539.33	622.40	24.84	0.172	0.570
Malondialdehyde (MAD) (nmol/l)	132.00	83.00	107.00	71.16	11.17	0.116	0.763

^*^SEM, standard error of the mean; *G1*, basal diet only; *G2*, basal diet + zinc oxide (40 mg Zn/kg diet); *G3*, basal diet + zinc lysine (40 mg Zn/kg diet); *G4*, basal diet + nano zinc oxide (40 mg Zn/kg diet)

Means in the same row with distinct superscripts are significantly different at *P* < 0.05

### IHC of NF-κB in Liver Tissues

**Fig. 4:**
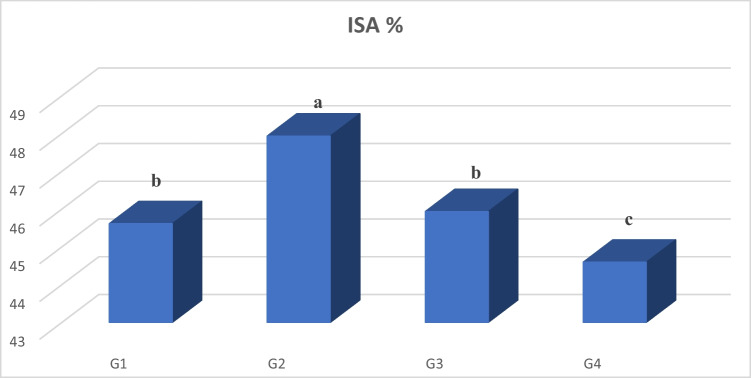
Immuno-stained area (ISA) % of nuclear factor kappa B (NF-*κ*B) in hepatocytes, means having the separate letters are significantly dissimilar from each other, *P* < 0.05

**Fig. 5:**
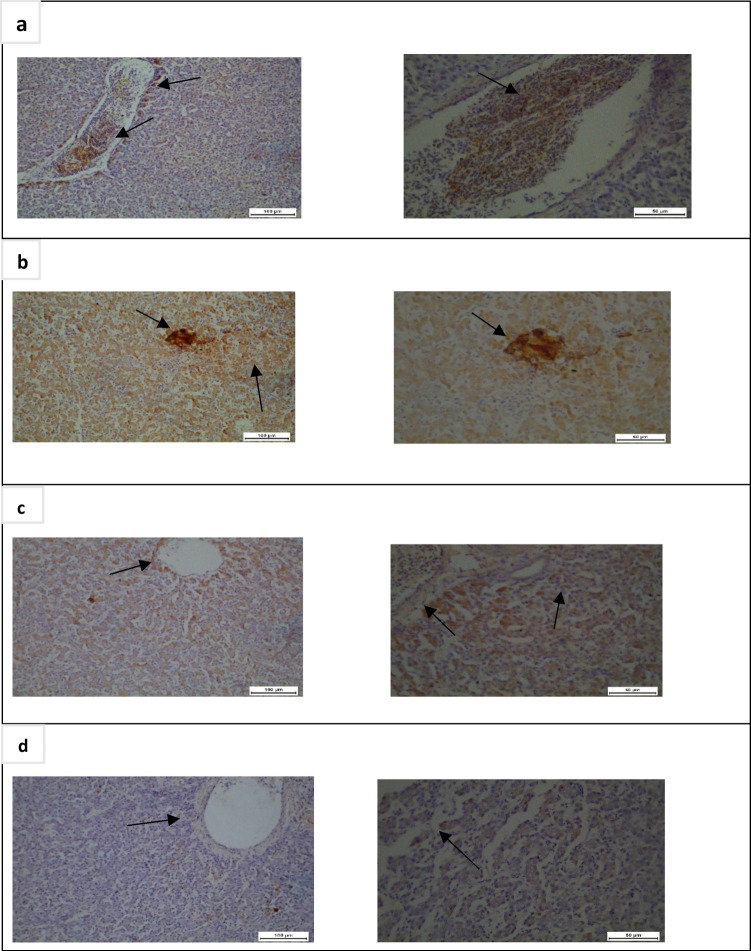
Immunohistochemistry of nuclear factor kappa B (NF-*κ*B) in the liver tissues of broiler chicks. Arrows refer to protein expression of NF-κB in hepatocytes, which positively stained brown in the liver cells. Bar = 50 and 100 μm. **a** G1 (basal diet only) showed diminished intracytoplasmic immune staining in hepatocytes. **b** G2 (basal diet + zinc oxide (40 mg Zn/kg diet) displayed strong intracytoplasmic immune staining in hepatocytes. **c** G3 (basal diet + zinc lysine (40 mg Zn/kg diet) showed diminished intracytoplasmic immune staining in hepatocytes. **d** G4 (basal diet + nano zinc oxide (40 mg Zn/kg diet) showed scanty intracytoplasmic immune staining in hepatocytes

The immune reactivity for NF-κB as well as immuno-stained area (ISA) % were linearly increased (*P* < 0.05) in the ZnO group than in those of the G1, G3, and G4. On the other hand, the NF-κB immunostaining intensity was linearly (*P* < 0.05) diminished in the liver of nano zinc oxide compared to other groups (Fig. 4). Tissue sections of the liver from G1 and G3 demonstrated low expression of NF-κB (Fig. 5a and c), whereas, tissue sections of the liver from G2 shown in Fig. 5b revealed overexpression of NF-κB. In contrast, the section of the liver from G4 shown in Fig. 5d revealed significantly very low expression of NF-κB staining.

## Discussion

The improvement of nano zinc oxide (NZnO) supplementation at the level of 40 mg Zn/kg diet on BW, BWG, FCR, and FE could be attributed to its characteristics. As, NZnO is very exciting because nano particulates show novel unique abilities such as shape, size, great surface area, high catalytic efficacy, and strong adsorbing capability [37]. Because of their small size, they are easier to take up by the gastrointestinal tract (GIT) mucosa, so they are more active than the ZnO at lower doses [38]. Owing to the permeability of NZnO, it can lessen GIT reactions and increase medicine absorption [39]. Also, Ibrahim et al. [40] reported that ZnO nanoparticles enhanced Zn retention, antioxidant status, and metabolism of broilers, which resulted in better performance. Furthermore, Hafez et al. [41] indicated that the performance development may be due to the ability of NZnO to raise the absorptive capacity of the intestine as it increases villi length and width, mucosal length, and crypt depth. This positive effect may be due to the role of NZnO in raising intestinal absorption ability by increasing mucosal efficiency. All of these characteristics magnify the NZnO functions and lead to improving the general health status and, accordingly, the growth performance parameters of birds. Moreover, the improvement in FCR and FE could be due to zinc-promoted feed utilization through participating in the metabolism of carbohydrates, lipids, and proteins [42]. The same findings were recorded by Zhao et al. [43], who stated that proper levels of nano zinc oxide (20 and 60 mg/kg diet) could improve body weight and weight gain compared with 60 mg/kg ZnO. Furthermore, Ahmadi et al. [44] used nano zinc oxide (30 to 90 mg/kg diet), Fathi et al. [45] used nano zinc oxide (20 mg/kg diet), and El-Katcha et al. [24] used nano zinc oxide (60, 45, or 30 mg/kg).. They reported that nano zinc oxide significantly enhanced (*P* < 0.05) the BW and BWG of broiler chickens. Our data also agreed with Lina et al. [46], Akhavan-Salamat and Ghasemi [47], and El-Haliem et al. [48], who confirmed that the feed consumption of broilers decreased significantly at the level of 40 mg/kg diet of nano zinc oxide (*P* < 0.05), and the feed/gain (F/G) was significantly improved (*P* < 0.01) at the same level on 42 days of feeding.

In contradiction to our finding Ramiah et al. [49] cited that ZnO-NPs at the level of 40, 60, and 100 mg/kg diet did not have any effect on the body weight of the broiler chickens compared to the 60 mg/kg diet of zinc oxide as control. El-Haliem et al. [48] also found that no significant differences were detected between experimental groups (100, 80, 60, 40, and 20 mg NZnO/kg of diet) in average values of final live body weight, total body weight gain compared to control diet that contained 100 mg zinc oxide /kg.. Besides, Eskandani et al. [23] declared that 30, 50, 70, and 90 mg Zn from Zn-NPs had no significant effect on average WG, average FI, and FCR during the rearing period of broiler chicks. The differences among studies might be associated with the size and chemical form of zinc, the quantity of zinc in the basal diet, supplemental level, and the length of the experimental period and rearing conditions.

In addition, our results showed that zinc lysine (G3), 40 mg Zn/kg diet, achieved an improvement in final body weight and cumulative weight gain. Many beneficial effects originated when organic Zn was offered to broilers, including growth rate enhancement [50]. This enhancement of zinc lysine in BW and BWG could have been due to the fact that organic Zn sources have better bio-availability [51]. According to reports, amino acid chelates have significantly better absorption rates from the GIT, via peptide or amino acid transport systems, due to their chemical structure [52]. Moreover, the high bioavailability of chelated Zn sources causes the excretion of microelements into the environment to be restricted, which permits improved absorption and utilization of nutrients without compromising the performance of the animals [53]. This is in a match with Liu et al. [54] cited that, as compared with Zn sulfate, broilers received diets supplemented with Zn proteinate (10, 20, 40, or 80 mg of Zn/kg diet) had higher average daily weight gain values (*P* = 0.106). Besides, Chand et al. [55] reported that the body weight was significantly higher (*P* < 0.05) in OZ-50 (50 mg organic zinc/kg diet), displaying the effectiveness of organic Zn. Different trends were reported by other researchers. Studies with organic zinc by Hudson et al. [56] indicated that the growth performance of broilers was unaffected by dietary organic zinc supplementation in excess of NRC (1994) recommendations of a 40 mg/kg diet. Also, body weight gain was not significantly affected (*P* ≤ 0.05) by organic zinc (Bioplex Zn) supplementation at the level of 15, 30, 45, or 60 ppm to a basal diet in the broiler [57]. The lack of response in the performance parameters among studies could be due to varied doses and different forms of zinc used in broiler diets.

Our data revealed that ZnO (G2) at a level of 40 mg Zn/kg diet has no significant effect on final body weight and cumulative weight gain compared to the control group. This is in a match with that confirmed by Wang et al. [58], who said that growth performance of broilers was unaffected by dietary inorganic zinc supplementation in excess of NRC (1994) recommendations of a 40 mg/kg diet. Yang et al. [59] evaluated the supplementation of the broiler's diet with Cu, Fe, Zn, and Mn as a conventional inorganic sulfate salt. They stated that there was no significant difference noted in ADG among treated groups. But the findings of the present study contradict those of other researchers. Lai et al. [60] confirmed that birds that obtained a 40 mg Zn/kg diet as zinc oxide under high-temperature conditions were sufficient for the optimum growth performance of the birds. Ezzati et al. [61] clarified that dietary Zn significantly enhanced daily body weight and weight gain when the diet was supplemented with 100 ppm Zn sulfate. The disagreement of our study with the other studies may be due to the amount of zinc in the basal diet or to the amount and sources added. Furthermore, other dietary ligands such as phytate combine insolubly with zinc and prevent it from being absorbed [57].

Our findings showed that zinc lysine and zinc oxide displayed significantly the best results in the FCR and FE with significantly lower feed intake (*P* < 0.05) compared to chicks in G1. The current results were also consistent with those obtained by Jahanian et al. [62] cited that increasing the Zn level from 80 to 120 mg/kg diet either from inorganic zinc (zinc oxide and zinc sulfate) or organic zinc (zinc–methionine, zinc–lysine, and zinc acetate) forms decreased the average feed intake (*P* < 0.001). Broilers that are fed 60 or 45 mg zinc polysaccharide complex/kg diet had an improved FCR. Also, using organic zinc reduced total feed intake [24]. In contrast, the progressive addition of zinc in organic form [57] or inorganic form [63], and in a combination of both as a complex form [64] to the basal diet did not affect the feed efficiency of broilers. Iqbal et al. [65] showed that zinc supplementation in broilers' diets, involving two Zn levels (40 and 80 mg/kg diet) in form of Zn propionate and Zn sulfate had no significant effect on FI, total BW, and FCR. The potential of zinc to enhance digestibility, FCR, and FE in broilers has been highlighted. The recorded enhancement may be due to zinc’s ability to activate the pancreas to release digestive enzymes, thereby improving nutrient digestibility and feed utilization [66], leading to a raised ADG. Also, zinc participates in many biochemical processes, and the observed performance development in the present study may be elucidated by the fact that the supplemented Zn level met the requirements of enzymes that have a crucial role in the synthesis of DNA, RNA, and body protein [67]. These positive effects were also recorded in G4 but were more prominent with nano zinc oxide than in G2 and G3 due to the higher bioavailability of nanoparticles.

Moreover, nano zinc oxide (G4) linearly increased the performance index, and European Efficiency Index which was parallel to the improved live body weight and FCR in the nano zinc oxide supplemented group. The high performance index could be due to better effects of zinc on digestion and utilization of nutrients, as well as greater bioavailability of zinc nanoparticles [68]. EEI inversely depends on FCR in the used equation so it may be co-approved that, improved FCR due to dietary nano zinc oxide at a level of 40 mg Zn/kg reflexed on significantly increased EEI at 35 days of age. As well as, nano zinc oxide linearly gave the best results in PER during finisher phase. Our data was confirmed by the findings of other studies. NZnO at 20, 40, and 60 mg/kg linearly improved the crude protein, crude fiber, and ether extract nutrient digestibility of broilers compared to the non-treated group [69]. This effect could be attributed to the improvement of enzymes' activity induced by zinc [53]. Furthermore, ether extract digestibility and zinc absorption were significantly improved (*P* < 0.05) in chickens that received the diet supplemented with 50 ppm of NZnO compared to those in the non-treated group [40].

To farther understand the mechanism of the recorded improved growth performance, we examined the level of mRNA transcripts of insulin-like growth factor (IGF-I). The rise in the number of mRNA copies of IGF- I expected reflects the enhanced growth performance of broilers. Moreover, IGF-I is one of the promising candidate marker genes for the development of the growth rate of chickens [70]. The addition of nano zinc oxide (G4) at a level of 40 mg Zn/kg diet linearly increased mRNA expression of IGF-I in the liver tissues of broilers, followed by the G3 then G2 that supplemented with zinc lysine and ZnO, respectively. The high bioavailability of nano ZnO and organic Zn indicates that more Zn was taken through the body and not only deposited in bone, but affected cellular function responsible for hormones and growth factor secretions. Additionally, Zn augmented the growth factor synthesis, as IGF-I, and influenced the action of calcium-regulating hormones [71]. Likewise, Zn plays an essential role in the formation of insulin through its enzyme systems [72]. It was stated that the anabolic impact of IGF-I on osteoblasts is boosted by Zn [58]. Keeping the above facts in view, our results regarding the enhanced effects of nano zinc oxide (G4), then zinc lysine (G3), then zinc oxide (G2) at a level of 40 mg Zn/kg diet on growth performance parameters of broilers were confirmed by the findings of increased mRNA expression of IGF-I in liver tissues, respectively. The present results are confirmed by those conveyed by Tomaszewska et al. [73], who explored that the concentration of insulin-like growth factor I of broilers was enhanced only in the zinc glycine supplied group at a level of 50 ppm compared to the control group. Ibrahim et al. [40] elucidated that the dietary organic Zn and/or nano ZnO significantly increased (*P* < 0.05) mRNA expression of IGF-I gene in broilers. Moreover, the mRNA expression of IGF-I gene tended to be increased in nano ZnO supplemented group than in Zn-mix and organic groups. Likewise, Zhang et al. [74] indicated that NZnO at a level of 40: 160 mg/kg linearly increased final BW (42 d) and serum IGF-I levels of broiler chicks.

Meat pH, water-holding capacity, color, texture, and drip loss are critical characteristics to consider when assessing the quality of meat because it has an impact on how consumers will rate it and how much they will pay. In our experiment, breast meat has a pH value of 5.56–6.83 which nearly complies with the typical pH values (about 5.3–6.5) [75] and the lower breast meat pH value reported in nano zinc oxide treatment. Muscle pH plays an essential role in the rate of microbial decomposition [76]. At the same refrigerated conditions, the high pH muscle creates conditions that make dark-colored fillets more vulnerable to bacterial decomposition than light-colored fillets (low pH muscle) [77]. Hence, the breast meat of the supplemental NZnO (40 mg Zn/kg diet) group is expected to have increased shelf life in an aerobic condition of storage. Besides, the total glycogen amount in muscle before slaughter and the rate of lactic acid formation after slaughter determine the pH of broiler meat [78]. As, NZnO positively affects the growth rate of birds by contributing to the metabolism of carbohydrates, lipids, and proteins [42]. Most meat quality traits, including meat colour, ERV, hardness, and drip loss, are strongly related to muscle pH [79]. Expressible release volume (ERV) is known as the amount of total meat water that can be expressed by applying force. In general, a higher ERV means that a greater percentage of the water is held more loosely, which results in a lower WHC [80]. The significantly highest ERV% (lowest WHC) in breast muscle that was observed in nano zinc oxide and zinc lysine group, was parallel with a lower pH value. The meat colour is a quality index, which denotes its freshness for the customer [81]; Moreover, Zn's ability to bind to myoglobin and boost its oxygenation enables the preservation of meat's colour. Additionally, zinc acts as an antioxidant, inhibiting mitochondrial respiration and reducing the generation of free radicals, which helps preserve the colour of meat [82]. Water-holding capacity (WHC) and a* values were significantly correlated with muscle pH and WHC, respectively [83] as shown in our study. Data from our study are parallel to those of Mahmoud et al. [84], who found that the values for meat colour in chickens offered diets containing 40 ppm of ZnO-NPs were significantly lower *(P* < *0.05)* than in the control group. As well, different Zn treatments (ZnSO_4_, Zn amino acid complex, and NZnO) did not show any effect on breast meat b* values and drip loss *(P* > *0.05).* It was sustained that the pH of breast muscle after 24 h was significantly higher in the non-treated group compared with other Zn treatment groups *(P* < *0.05)* [23]. The observed non-significant effect of different zinc sources on hardness could be attributed to the fact that in broilers, many dietary factors affect meat texture (tenderness and hardness), such as age, nutrition, and sex [85]. Also, the recorded pH in the present study was within the low range, but this did not disturb dependent technological features including drip loss (24 h), all within the perceived range. This directs that the breast meat from each treatment group had no issues with quality. This agrees with Kakhki et al. [86], who cited that different levels of organic Zn and α-tocopheryl acetate (α-TOA) supplementation and their interactions did not impact the drip percentage of the breast and thigh muscles of broilers. Zakaria et al. [64] illustrated that meat quality of breast showed a significant improvement *(P* < *0.05)* in shear force (tenderness), indicating juicier meat in chickens receiving a diet supplemented with 80 mg/kg diet of ZnO plus 42 mg of an organic Zn-amino acid complex.

The total antibody titer recorded on the 35^th^ day showed a numerical increase in the zinc lysine (G3) and nano zinc oxide (G4) groups. Virden et al. [87] declared that Zn contributes to the function of superoxide dismutase, which is vital for the integrity of macrophages and heterophils that stimulate higher immune responses. Additionally, Zn is necessary for thymulin, a thymic hormone that controls the development of T lymphocytes and the activation of B lymphocytes [88]. Birds that received diets added with a highly available zinc source (NZnO and zinc lysine) might have stimulated thymulin activity and thus elicited immune reaction through augmented T lymphocyte maturation and B lymphocyte activation by T-helper cells [61]. These results were in accordance with Mahmoud et al. [84], who elucidated a numerical increase in the antibody titer against the ND vaccine at a level of 10 ppm ZnO-NPs. Additionally, Sahoo et al. [89] mentioned that dietary NZnO might have prompted a better immune response even at lower physiological limits. As well, the dietary concentrations of organic zinc (40 mg/kg diet) did not have an impact *(P* > *0.05)* on the immunological responses (antibody titers of ND) in broiler chicks raised under heat stress conditions at 21 days of age [15]. In contrast, Smith [90] mentioned that birds provided diets with zinc poly amino acid complex had significantly increased levels of total antibody titers. Soni et al. [91] clarified that organic zinc supplementation to broiler breeders significantly increased cellular immunity and antibody production. The lack of significant findings in immune response in our study could result from the variation in the level of the organic zinc used. The significant response detected in the previous study was possible a result of using comparatively larger Zn levels (up to 181 mg/kg) than what was used in our work (40 mg Zn/kg).

Concerning the results of the present study, which revealed a numerical elevation in the serum SOD and numerical reduction in the serum MDA in the nano zinc oxide supplemented group, these results came in harmony with Fathi [92], who showed that higher concentrations of NZnO (40 mg/kg) had no significant effect on serum copper-zinc superoxide dismutase (Cu–Zn-SOD) activity of chickens. El-Katcha et al. [24] said that zinc polysaccharide complex was not associated with a significant influence on blood serum MDA concentration, while nano zinc supplementation non-significantly reduced blood serum MDA concentration compared to chickens receiving inorganic zinc-supplemented diet. In addition, Zn-Met and NZnO at a dose of 40 mg/kg of diet could positively affect the antioxidant activity of broilers raised under high ambient temperature [47]. Our results were inappropriate with those obtained by Ahmadi et al. [93], who sustained that total antioxidant capacity (TAC) and superoxide dismutase had significantly increased *(P* < *0.05)*, as well malondialdehyde decreased *(P* > *0.05)* in chicks fed a diet containing nano zinc oxide at a level of 60 or 90 ppm in comparison to other treatments. Abdel-Monem et al. [94] and Dukare et al. [95] revealed that an 80 mg NZnO/kg diet resulted in a significant elevation in SOD and TAC and a reduction of lipid peroxidation of chicken.. The non-significant conclusion of NZnO on the antioxidant activity might be associated with the level of zinc in the diet and might be due to the use of relatively higher concentrations of NZnO than used in previous studies. Many investigations in different livestock have demonstrated the critical role of zinc in lipid peroxidation reduction and antioxidant defense improvement [96, 97]. This is attributed to Zn being a pivotal constituent in copper-zinc superoxide dismutase (Cu–Zn-SOD). Cu–Zn-SOD participates in the cellular removal of reactive oxygen species (ROS) and free radicals [98]. Powell [82] reported that Zn is regarded as a cofactor of more than 300 enzymes and affects oxidative reactions. Zinc is also a powerful inducer of metallothionein (MT), that scavenges ROS and could serve as reduction–oxidation sensor or active signaling switch in cells [99]. MDA is a crucial indicator of lipid peroxidation and oxidative damage produced by ROS [100]. So, high MDA values were correlated with high oxidized lipid values. Zago and Oteiza [101] also elucidated the role of zinc, which is vital in antioxidative defense, in the reduction of lipid oxidation processes. Previously, Powell [82] clarified that Zn decreases MDA values, which shows that Zn is considered an antioxidant factor, as zinc diminishes lipid peroxidation in the cell.

NF-*κ*B is the principal transcription factor in tissue damage and inflammation [102]. It also is necessary for regulating DNA transcription and is involved in cell response to numerous stimuli, including free radicals [103]. It was suggested that ROS may serve as important secondary mediators responsible for activating NF-*κ*B in response to a variety of stimuli because of its strategic relation between oxidative stress and inflammation [104]. In commercial production, broilers are normally subjected to frequent stress stimuli such as climatic stress, environmental stress (litter, light, and ventilation), nutritional stress, physiological stress (fast growth and process of maturing sexually), physical stress (catching and transport), and psychological stress [105]. As a result of these various stressors, the first responder is the NF-B complex protein, which recruits other proteins such as RNA polymerase and alters cell function [106]. Birds must be adapted to external stimuli. This process of adaptation impairs development, reproduction, and health by releasing hormones and requiring the redistribution of physiological reserves, such as protein and energy. [107]. According to our findings, nano zinc oxide successfully reduced the expression of NF- κ B (a stress indicator), which may indicate that nano zinc oxide supplementation reduces the inflammatory response. So, the energy and nutrients used for inflammatory responses will be secure for productive purposes. On the parallel side, our data revealed the best achievements of NZnO on the broiler’s growth performance parameters at 35 days of the experiment. Our results were in harmony with those found by Kim and Jeong [108] reported that ZnO-NPs (0.1, 1, and 10 μg/mL) prevented I _*κ*_ B _*α*_ phosphorylation and degradation which inhibited the nuclear translocation of nuclear factor- κβ. In addition, ZnO-NPs encouraged the expression of A20, a negative regulator of NF-*κ*B and zinc finger protein. They also demonstrated that ZnO-NPs provides a possible approach for combating inflammatory disorders. In vitro trials have shown that Zn reduces NF-*κ*B and its specific gene upregulation, such as IL-1β and TNF-α, while increasing anti-inflammatory gene expression, A20 and PPAR-a, the two zinc finger proteins [109]. Unfortunately, scarce literature evaluated the effect of different zinc sources on IHC of NF-κB in broiler chickens. Meanwhile, Xie et al. [110] indicated that replacement of antibiotics with *Enteromorpha prolifera* polysaccharide–zinc (EP–Zn) in diets can increase the immune response, diminish the expression of NF-*κ*B and the release inflammatory factors, preserve the integrity of the GIT barrier, and decrease the rate of diarrhea in weaned piglets.

It could be concluded that the best achievements of nano zinc oxide, 40 mg Zn/kg diet, on the growth performance parameters and elevated mRNA expression of IGF-I. The breast meat of the NZnO supplemental group is expected to have an increased shelf life in aerobic storage. NZnO exhibits a better immune status and alleviates oxidative stress and inflammatory response in broilers. These positive effects were also observed in the zinc lysine then zinc oxide groups but were more pronounced in the nano zinc oxide group due to the higher bioavailability of nanoparticles. Therefore, we recommend using nano zinc oxide (NZnO) at a level of 40 mg Zn/kg diet in mineral premix for broiler feeding regimens.

## Data Availability

This article includes the data generated during this experiment.
